# A general prediction model for the detection of ADHD and Autism using structural and functional MRI

**DOI:** 10.1371/journal.pone.0194856

**Published:** 2018-04-17

**Authors:** Bhaskar Sen, Neil C. Borle, Russell Greiner, Matthew R. G. Brown

**Affiliations:** 1 Department of Computing Science, University of Alberta, Edmonton, AB, Canada; 2 Alberta Machine Intelligence Institute, AB, Canada; 3 Department of Psychiatry, University of Alberta, Edmonton, AB, Canada; McGill University, CANADA

## Abstract

This work presents a novel method for learning a model that can diagnose Attention Deficit Hyperactivity Disorder (ADHD), as well as Autism, using structural texture and functional connectivity features obtained from 3-dimensional structural magnetic resonance imaging (MRI) and 4-dimensional resting-state functional magnetic resonance imaging (fMRI) scans of subjects. We explore a series of three learners: (1) The LeFM_*S*_ learner first extracts features from the structural MRI images using the texture-based filters produced by a sparse autoencoder. These filters are then convolved with the original MRI image using an unsupervised convolutional network. The resulting features are used as input to a linear support vector machine (SVM) classifier. (2) The LeFM_*F*_ learner produces a diagnostic model by first computing spatial non-stationary independent components of the fMRI scans, which it uses to decompose each subject’s fMRI scan into the time courses of these common spatial components. These features can then be used with a learner by themselves or in combination with other features to produce the model. Regardless of which approach is used, the final set of features are input to a linear support vector machine (SVM) classifier. (3) Finally, the overall LeFM_*SF*_ learner uses the combined features obtained from the two feature extraction processes in (1) and (2) above as input to an SVM classifier, achieving an accuracy of 0.673 on the ADHD-200 holdout data and 0.643 on the ABIDE holdout data. Both of these results, obtained with the same LeFM_*SF*_ framework, are the best known, over all hold-out accuracies on these datasets when only using imaging data—exceeding previously-published results by 0.012 for ADHD and 0.042 for Autism. Our results show that combining multi-modal features can yield good classification accuracy for diagnosis of ADHD and Autism, which is an important step towards computer-aided diagnosis of these psychiatric diseases and perhaps others as well.

## 1 Introduction

Statistical machine learning methods have recently permeated disciplines such as Psychiatry, which specializes in the diagnosis and treatment of neuropsychiatric disorders [1]. The availability of large scale neuroimaging datasets has encouraged researchers to develop computer-aided tools and procedures for understanding the human brain and its disorders. Some studies use structural Magnetic Resonance Imaging (MRI) scans, which provide a non-invasive technique for obtaining a volumetric image of the brain anatomy, while others use functional MRI (fMRI) scans, which measure brain activity by detecting fluctuations in the Blood Oxygenation Level Dependent (BOLD) signal over time.

Many neuroscientists are seeking ways to use such MRI and/or fMRI data to detect brain function disorders, such as Autism and Attention Deficit Hyperactivity Disorder (ADHD). While significant work has been done in *association studies* for finding discriminative group-level characteristics for ADHD [2–4] (resp., Autism [5, 6]), we are seeking a general learning tool that, when given relevant data, can learn an accurate *predictive model*, which can predict whether an individual subject has a particular disease. This paper explores *biologically naive* ways to learn combinations of state-of-the-art structural texture features (from structural MRI scans) and functional connectivity (from fMRI scans) to predict whether a subject has ADHD (resp., Autism). Specifically, our learned models use recently-developed unsupervised feature learning from images and independent component analysis of fMRI for prediction of the diseases.

From brain images, the structural textures provide information about the spatial arrangements of voxel intensities in 3 dimensions, which in turn can describe neurological aspects of a subject’s brain. On the other hand, functional connectivity captures patterns of deviations from statistical independence between the time signals at distributed, and often spatially remote, neuronal regions [7–9]. Deviations from statistical independence are generally taken to indicate dynamic coupling and can be measured, for example, by estimating the correlation, independent components, etc.

As a first step towards creating a generalized prediction tool for ADHD/Autism, we experiment with 3-D texture based models that describe structural arrangements of the brain by learning texture features from MRI scans. These features are then used by our LeFM_*S*_ learner. To use the fMRI data, we experiment with four different source separation techniques, that each decompose the scans into spatio-temporal components that are common across the subjects. (Each component specifies a timecourse. For each each, for each component, a spatial map is created based on how simliar each voxel’s time course is to the component time course. See Methods for details.) This, in turn, allows each subject’s fMRI scan to be described as a weighted sum of the common spatio-temporal components, with the subject’s weights then serving as features for our model. Our LeFM_*F*_ learner uses these fMRI-derived features to produce classifiers designed to predict whether a patient has a particular disease. Finally, our learner LeFM_*FS*_ uses both sets of features (structural and functional) to produce a classifier designed to predict if a patient has a particular disease.

Here, we use two publicly available, multi-site datasets, ADHD-200 and ABIDE (http://fcon_1000.projects.nitrc.org/indi/abide/), for developing and then testing our models. Since the publication of the ADHD-200 dataset, many researchers [10–13] have explored ways to improve the prediction accuracy of ADHD using this data; see also [14] for Autism. However, even the best prediction results are not at the level of being clinically useful.

The main contribution of this work is our approach to feature extraction—*i.e*., a single method to obtain features, by applying digital image processing algorithms to structural and functional MRI scans, that can be used in learned models to accurately distinguish between ADHD versus Healthy (resp., Autism versus Healthy) subjects. In particular,

1. While standard independent component analysis (ICA)-based source separation is used primarily in analyzing group-level activation differences in fMRI analysis, we devise a novel extension, and algorithm, that is applicable to prediction studies (Section 2.5). We show that the resulting LeFM_*F*_ algorithm, with its learned combination of these learned features, out-performs other fMRI-based algorithms for ADHD/Autism prediction, on these datasets.
2. In addition to using ICA-based source separation in the context of a predictive study, we introduce the approach of using multiple decorrelation [15] for the purpose of fMRI source separation and compare it to three other commonly used source separation algorithms. Here, fMRI volumes are modeled as non-stationary signals and source separation based on second-order criteria is used to separate out common spatial activation maps and subject-specific time courses.
3. Combining texture-based features learned from MRI and features extracted using fMRI source separation, we improve the prediction model accuracy for ADHD to 0.673 (compared to 0.661) and Autism to 0.643 (compared to 0.619).
4. Our application of image processing algorithms for structural/functional MRI data suggests that *biologically naive* features can lead to effective classifiers of mental disorder (ADHD/Autism), suggesting this approach should be further investigated for these, and other disorders.

## 2 Materials and methods

**Fig 1:**
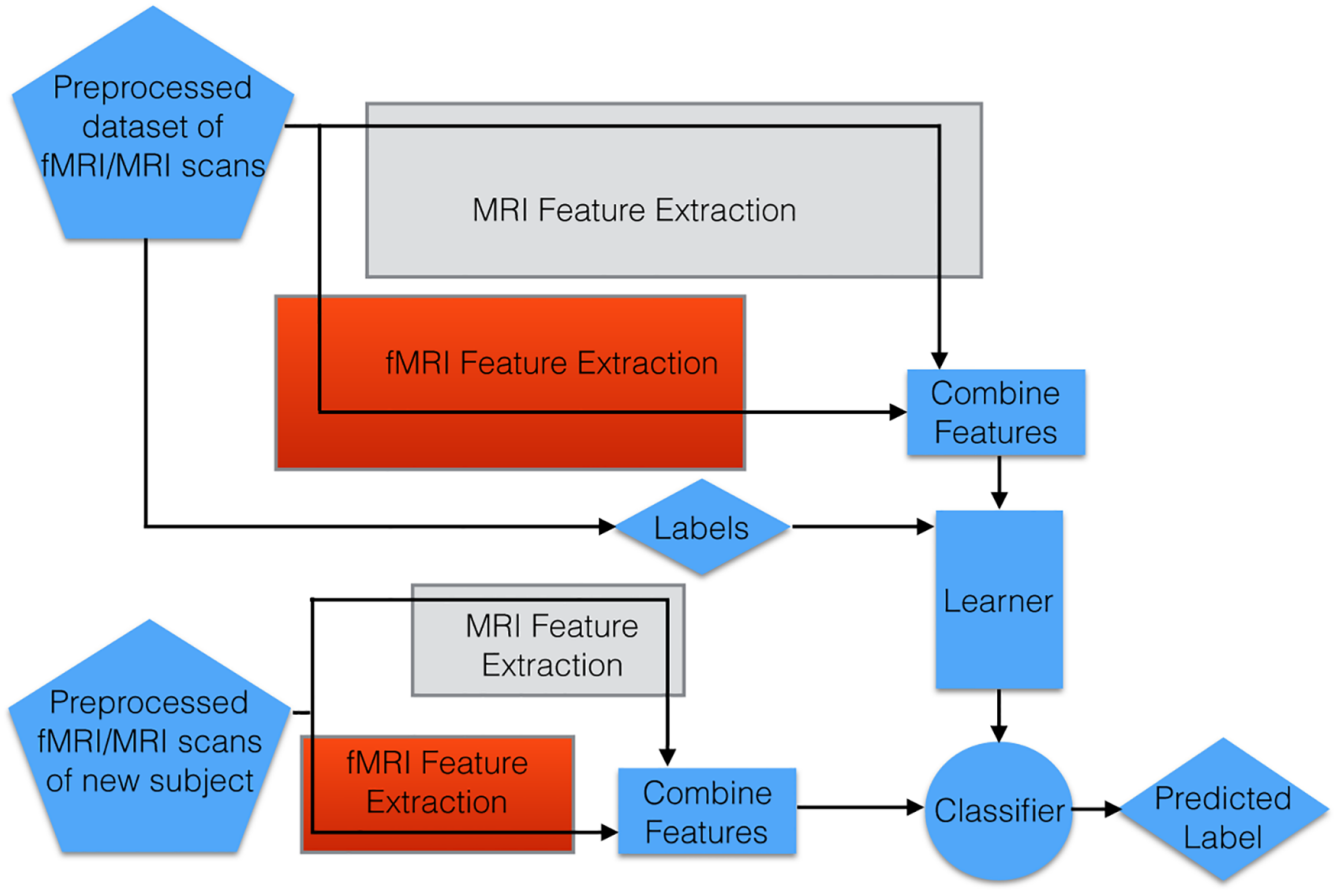
Overall pipeline, including feature extraction steps, for each of our methods: LeFM_*SF*_ uses all of these steps (as it uses both MRI and fMRI data); LeFM_*S*_ does not use the red “fMRI feature extraction” box; and LeFM_*F*_ does not use the grey “MRI feature extraction” box. In each case, in addition to the learner that utilizes the combined features, our system also learns the most effective features from the MRI data (grey box) and from the fMRI data (red box); those features are used to pre-process the test data (see boxes of the same color).

This section presents the overall process of the diagnostic system; see Fig 1 We first describe the datasets (Section 2.1) and evaluation criteria (Section 2.2). Then we outline the preprocessing pipeline in Section 2.3. The remaining sections summarize diagnostic methodology from MRI scans (LeFM_*S*_; Section 2.4), from fMRI scans (LeFM_*F*_; Section 2.5), and from combined imaging features (LeFM_*SF*_; Section 2.6).

### 2.1 Datasets

We used one multi-site dataset for producing, then evaluating, each model: ADHD-200 (including 8 sites) and ABIDE (including 17 sites). Each of the datasets included a structural scan (high resolution, for a single time point), and also one or more resting-state functional scans for each of the subjects. The spatial resolution of the structural MRI scans was 1*mm* × 1*mm* × 1*mm*. In these *resting state* functional scans, the subject did not perform any explicit task. That functional scan included between 76 to 261 time points for each ADHD-200 subject and between 82 and 320 time points for each ABIDE subject. Different subjects were scanned with different temporal resolutions: ranging from 1.5 seconds through 3 seconds in the ADHD-200 dataset, and from 1 seconds through 3 seconds in the ABIDE data. The field strength of the MRI scanners varied from 1.5T to 3T. Each data collection site used its own scanner(s) and its own MR scanning parameters. The demographics of subjects in these datasets can be found in Tables 1 and 2. More details are available at the ADHD-200 site (http://fcon_1000.projects.nitrc.org/indi/adhd200/) and ABIDE site (http://fcon_1000.projects.nitrc.org/indi/abide/).

**Table 1:** ADHD-200 data demographics. Site abbreviations: *Peking University (Peking), Kennedy Krieger Institute (KKI), NeuroIMAGE (NI), New York University (NYU), Oregon Health and Science University (Oregon), University of Pittsburgh (Pitt), Washington University in St. Louis (WashU)*.

	Peking	Brown	KKI	NI	NYU	Oregon	Pitt	WashU
Subjects	245	26	94	73	263	113	98	61
ADHD	130	26	33	50	163	71	9	0
Male/Female	174/71	9/17	64/30	43/30	171/92	61/52	53/45	33/28
Age Mean	11.7	14.54	10.22	17.64	11.45	0.10	15.08	11.47
Age STD	1.96	2.54	1.34	3.05	2.91	1.20	2.78	3.88

**Table 2:** ABIDE data demographics. Site abbreviations: *Carnegie Mellon University (CMU), California Institute of Technology (Caltech), Kennedy Krieger Institute (KKI), Ludwig Maximilians University Munich (LMU), New York University (NYU), Olin Institute of Living at Hartford Hospital (Olin), Oregon Health and Science University (Oregon), San Diego State University (SDSU), NeuroIMAGE (NI), Stanford University (Stanford), Trinity Centre for Health Sciences (Trinity), University of California, Los Angeles (UCLA), University of Leuven (Leuven), University of Michigan (UMich), University of Pittsburgh School of Medicine (Pitts), University of Utah School of Medicine (Utah), Yale University (Yale)*.

	CMU	Caltech	KKI	LMU	NYU	Olin	Oregon	Sdsu	
Subjects	27	38	55	57	184	36	28	36	
Autism	23	19	22	24	79	20	13	14	
♂/♀	23/4	32/6	48/7	48/9	154/30	31/5	24/4	30/6	
Age Mean	25.4	22.3	10.4	20	13.9	17.2	10	14.4	
Age STD	4.5	4.1	1.4	9.1	5.1	3.2	1.8	1.5	
	NI	Stanford	Trinity	UCLA	Leuven	UMich	Pitts	Utah	Yale
Subjects	30	40	49	108	64	145	57	101	56
Autism	15	20	24	62	29	68	30	58	28
♂/♀	24/6	17/3	19/5	52/10	24/5	59/9	25/5	51/7	26/2
Age Mean	29.5	9.5	16.6	12.7	21.4	13.8	17.9	24.5	12.4
Age STD	5.9	1.7	3.0	2.1	2.3	2.7	5.5	3.7	2.9

#### 2.1.1 ADHD-200

The ADHD-200 dataset is a multi-site combination of neuroimages taken from 8 sites (of which we use 7). It is partitioned into two disjoint sets (training and holdout), where the prediction model is learned from only the training set, and the learned model’s accuracy is measured on the holdout set. The data from the excluded site (Brown University) are not used because subject labels (ADHD vs. control) are not available for that site and we require labels for our machine learning algorithms. Thus, our training set consists of 776 resting state scans: 491 were taken from healthy controls and 279 from patients. To balance our training set, we used all 279 patients and selected 279 healthy controls evenly taken from all the sites as our training set [11, 12, 16], which means that the baseline classification accuracy for the training set is 0.50. The ADHD-200 competition hold-out dataset (excluding Brown University data) consists of 171 subjects: 94 healthy subjects and 77 ADHD cases (baseline accuracy 0.5497). We only use this set for evaluating the quality of the final model; *n.b*., it is not used in any way during the training process.

The ADHD-200 dataset also includes other non-imaging features for each subject, including gender, age, handedness, site of the imaging, IQ measure, etc. [17]. However, we only use imaging data for our experiments.

#### 2.1.2 ABIDE

The ABIDE dataset consists of 1111 scans, taken from 17 sites consisting of 573 healthy controls and 538 patients with autism. To evaluate each learning model, we use 800 subjects (70%) for model training and 311 subjects (30%) for hold-out testing. We use the same case/control ratio (0.5157) for both training and test set. The ABIDE dataset provides an array of non-imaging information which includes age, gender, handedness, various IQ scores, site of the imaging and eyestat (which indicates whether the person kept their eyes open or not during the scan). Again, we only use imaging data for our experiments.

### 2.2 Evaluation criteria

For each task, we use 5-fold cross validation (CV) [18] over the training set to set the parameters, based on accuracy. That is, each set of parameter values is tested with 5-fold cross validation on the training set, and the set of values producing the best average accuracy across all 5 folds of the training dataset is selected for final testing on the held out test dataset. We report the training set CV accuracy as well as performance measures of the learned model applied to the holdout set: accuracy, sensitivity, specificity and J-statistics (Jstat = sensitivity + specificity − 1).

We also used statistical tests to evaluate the hold-out results obtained by the various learners. For each dataset, we used a binomial test to compare our learners (LeFM_*F*_ and LeFM_*SF*_) to cross-validation baseline accuracies, to determine if each learner is significantly better than chance. We used two additional binomial tests to compare LeFM_*SF*_ to the previous state-of-the-art classifiers for ADHD-200 and ABIDE. Finally, four McNemar tests were performed to determine if the results of LeFM_*SF*_ were significantly better than those of LeFM_*S*_ or LeFM_*F*_ on the ADHD-200/ABIDE hold-out datasets.

### 2.3 Preprocessing pipeline

For preprocessing fMRI and MRI scans, we used SPM8 (http://www.fil.ion.ucl.ac.uk/spm/software/spm8/) and our own in-house MATLAB code. Preprocessing was identical to that used in Ghiassian *et al*. [13]. Preprocessing involved 6 steps:

1. 6-parameter rigid body motion correction of functional scans
2. Co-registration of functional scans to subject-specific structural scans to guide the spatial normalization step
3. Non-linear spatial normalization (parameter estimation and spatial transformation) of structural images to the MNI T1 template (http://imaging.mrc-cbu.cam.ac.uk/imaging/Templates)
4. Non-linear spatial normalization of previously co-registered functional image volumes (in step 2) to the MNI T1 template using warping parameters computed in the structural image normalization
5. Spatial smoothing of functional image volumes with 8mm full-width half-maximum (FWHM) Gaussian kernel
6. Z-normalization of each 3D volume’s intensities for structural and functional image to standardize the intensities of images scanned from different sites.

Note that only steps 2, 3 and 6 are applicable to processing the MRI images. For more details about the preprocessing steps, see Ghiassian *et al*. [13].

### 2.4 Building a model from structural MRI features, LeFM_*s*_

**Fig 2:**
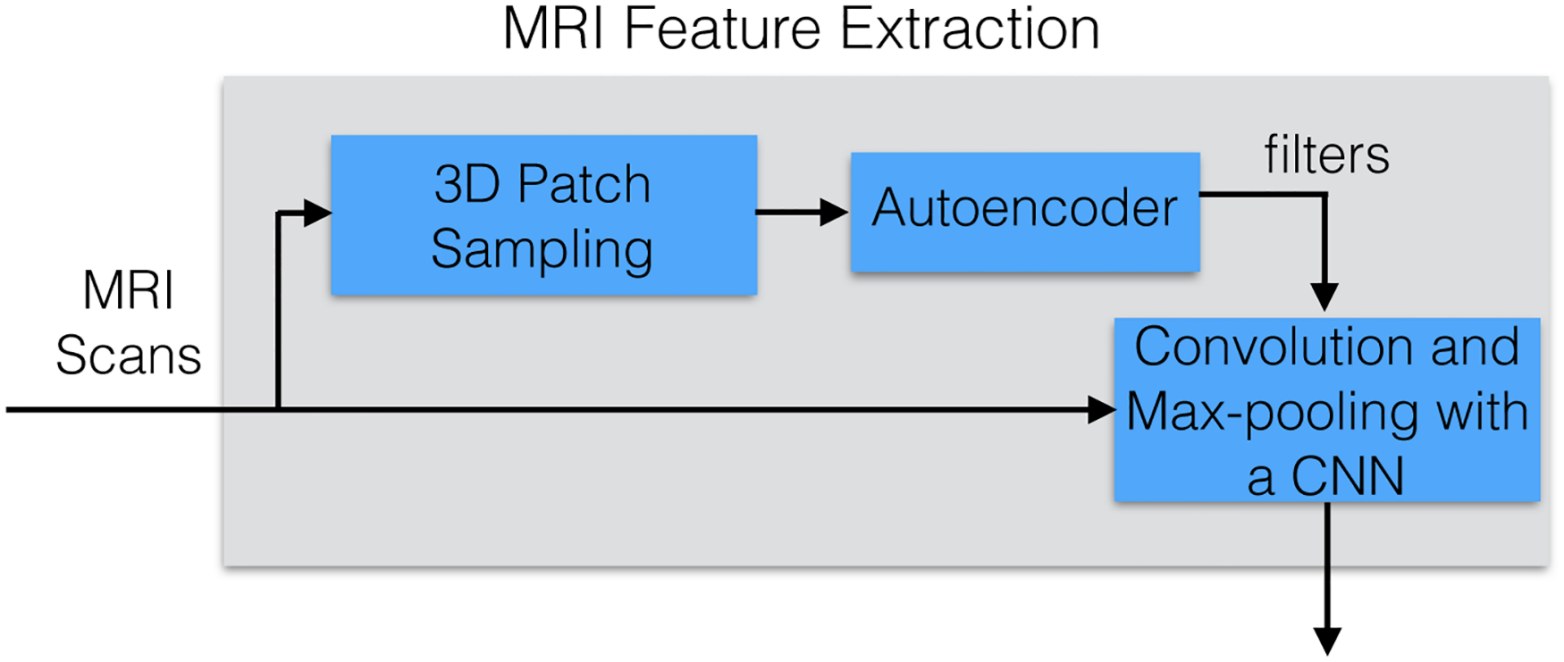
Process of extracting MRI features to be provided to a learning algorithm. This expands the “MRI feature extraction” box from Fig 1.

This section describes LeFM_*s*_, which extracts the usable features from structural MRI images, which are then provided to a learning algorithm; see Fig 2. Section 2.7 below describes the Learner, and how we set its hyperparameters. Here, we focus on the grey box, which does this preprocessing: this involves creating 3D patches by extracting all 5 × 5 × 5 cubes from the MRI image (Section 2.4.2), which are given to a sparse autoencoder that learns to encode a set of reduced representations (Section 2.4.3) of the patches producing filters (Section 2.4.1), which are then finally used in a convolutional neural network (Section 2.4.4) with convolution (Section 2.4.1) and max pooling to produce a final reduced feature set.

To understand this process better, we first define some common terms—Filters, Convolutions—that will be used later in this section. The rest of the section then describes the steps in Fig 2, in left-to-right order.

#### 2.4.1 Background: Filters, convolution

While our application deals with 3D images, this section explains the basic ideas using simpler 2D examples.

In image processing, filters are transformations that accentuate certain features within an image. Filters are generally defined on a neighborhood. For example, the 2D image filter

$$\begin{array}{c} h_{contrast}\;=\;[\begin{array}{ccc} 0 & \;1 & 0\\ 1 & -4 & 1\\ 0 & \;1 & 0 \end{array}] \end{array}$$ (1)

is defined on 3 × 3 neighborhood. Below, we view *h* as a function—here *h*_*contrast*_: {−1, 0, 1} × {−1, 0, 1} ↦ ℜ.

We then “convolve” a filter against an image. More precisely, a convolution is a mathematical operation on two functions—an image 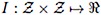 and a filter 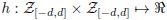 (where 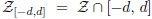 are the integers from −*d* through + *d*, inclusive)—to produce a third function that is typically viewed as a modified version of *I*(*x*, *y*), giving the overlap between the two functions. In case of 2-dimensional convolution, *I* is a 2D image (where *I*(*x*, *y*) is the intensity at position (*x*, *y*)), and *h*(*u*, *v*) is the filter that is convolved with the image—such as the *h*_*contrast*_ from Eq 1. The result of the convolution can be interpreted as the similarity measures between each pixel of the image and the filter. For 2D images, this result, at location (*x*, *y*), is defined as

$$\begin{array}{c} g\left(\;x,\;y\;\right)\;=\;\sigma (\sum_{(u,v)\in U}I\left(x-u,\;y-v\right)\;h\left(u,v\right)\;) \end{array}$$

where 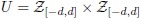 is the neighborhood over which the filter *h* is defined, and

$$\begin{array}{c} \sigma \left(t\right)=1/\left(1+e^{-t}\right) \end{array}$$ (2)

is the sigmoid function.

#### 2.4.2 3D patch sampling

The 3D patch sampling process converts our MRI image into a set of smaller 3D images. This is done by defining a patch size (in our case 5 × 5 × 5) and moving this cube across the image in all dimensions (step size is 1 voxel), extracting the contents of the cube at each point to create a 3D patch. This produces one such patch for every possible 5 × 5 × 5 cube that can fit within the original image—yielding approximately as many patches as there are voxels. Note that “adjacent” patches will overlap.

#### 2.4.3 Using a sparse autoencoder to generate filters

**Fig 3:**
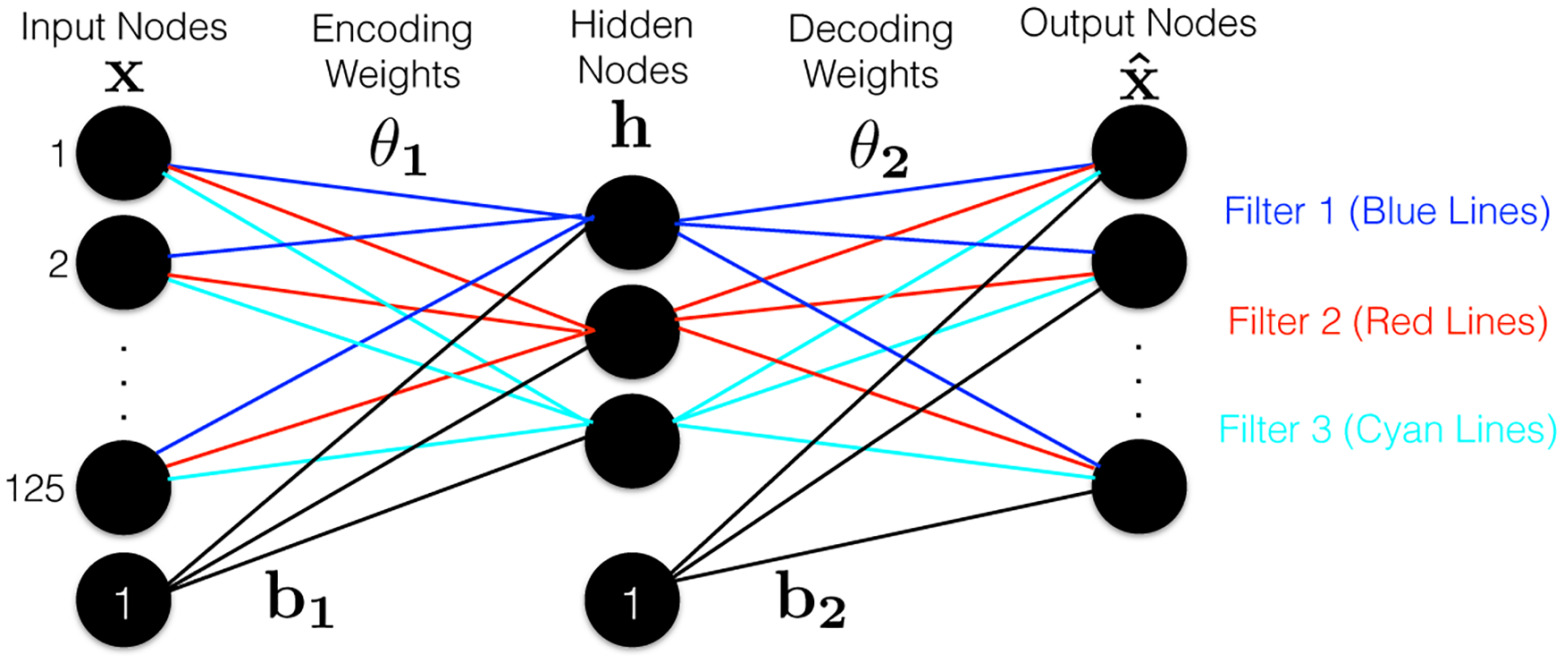
Autoencoder that tries to reconstruct the 5 × 5 × 5 patches (represented as a 125-D vector). For each of the *k* = 3 filters, there is a link (“Encoding Weights” *θ*_1_) from each input node to each hidden node, and then from each hidden node to each Output node (“Decoding Weights”, *θ*_2_); we show only a subset for clarity.

We then run a sparse autoencoder over the patches, to produce the filters required for convolution. To generate the filters for the convolutional neural network (CNN), we use a single layer unsupervised sparse autoencoder, that seeks to compress the 125-dimensional patches down to just 3-dimensions; see Fig 3. This produces 3 filters (based on the Encoding Weights); see left portion of Eq 1.

The sparsity aspect of the autoencoder encourages the network to learn different transformations in each of the nodes [19], which is meant to improve the robustness of the classifiers using the filters’ outputs as features. Formally, we seek the parameters [*θ*_**1**_, *θ*_**2**_, **b**_**1**_, **b**_**2**_] of the network (Fig 3) that minimizes:

$$\begin{array}{ccc} J_{D}\left(\theta ,b\right) & = & \frac{1}{2|D|}\sum_{x\in D}L\left(x,\hat{x}\right)\;+\;\beta \sum_{j=1}^{k}KL\left(\;\rho \;\|\;{\hat{\rho }}_{j}\right)\;+\;\lambda {\left\|\theta \right\|}_{2}\\ \hat{x} & = & \sigma (\theta_{2}h+b_{2})\\ h & = & \sigma (\theta_{1}x+b_{1}) \end{array}$$ (3)

where *D* ∈ ℜ^*m*×*n*^ is the data matrix, where each row is a data point **x** ∈ ℜ^*n*^, also **h** ∈ *R*^*k*^ is the hidden representation of the data (*i.e*., there are *k* nodes in the hidden layer), $\hat{x}\in R^{n}$ is reconstructed data, *L*(**a**,**b**) = ∑_*i*_(*a*_*i*_ − *b*_*i*_)^2^ is the squared loss, *σ*(*s*) is sigmoid function (Eq 2), *ρ* is sparsity parameter (*e.g*., *ρ* = 0.05), ${\hat{\rho }}_{j}=\;E\left[h_{j}\right]$ is average activation of the *j*^*th*^ hidden neuron, and

$$\begin{array}{c} KL\left(\;\rho \;\|\;{\hat{\rho }}_{j}\right)=\rho \log(\frac{\rho }{{\hat{\rho }}_{j}})\;+\;\left(1-\rho \right)\log(\frac{1-\rho }{1-{\hat{\rho }}_{j}}) \end{array}$$

is the Kullback-Leibler divergence. We used (internal) 5-fold cross-validation to select the hyperparameters *β*, λ and *ρ*, and solve Eq 3 using the L-BFGS package (http://users.iems.northwestern.edu/~nocedal/lbfgsb.html).

#### 2.4.4 Single layer unsupervised convolutional neural net

**Fig 4:**
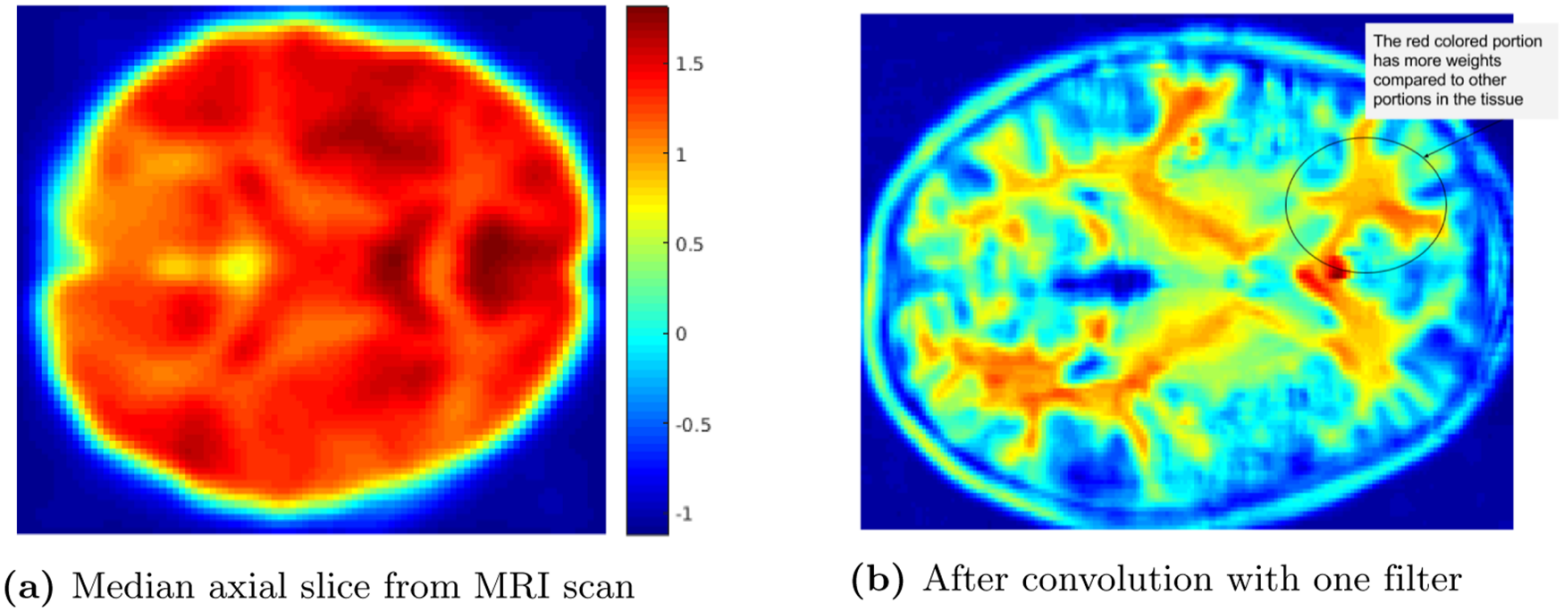
(a) Median axial slice from MRI scan and (b) the result after convolution with a filter.

After learning the *k* = 3 filters from the sparse autoencoder, the filters are then convolved throughout each entire MRI 3D image (size 79 x 95 x 68), producing a new 3D image (size 74 x 91 x 64) for each filter. (Fig 4 shows a 2D analogue of this.) As we use sigmoid activation functions *σ*(⋅), these features are a non-linear combination of voxels within the input image [20].

We then use *max-pooling* after the convolution step. Here, for each volume (produced by each filter), we divide the entire volume into disjoint and exhaustive 5 × 5 × 5 sub-regions, and then compute the maximum value of each sub-region. This step reduces the sensitivity of the feature map to various distortions, for example, by introducing translational invariance for the features within the max-pooling region. We then collect these values into a single vector—here of size 3 × (15 × 18 × 13) = 10 530. We then describe each subject with a single vector that is the concatenation of all *k* of these size-10,530 vectors. This is the input to the base learning algorithm; see Fig 1.

#### 2.4.5 Evaluating the model produced by LeFM_*s*_

To evaluate the LeFM_*s*_ algorithm, we used 5-fold CV on the training set with internal 5-fold CV to obtain hyperparameters that maximize CV accuracy, and then (after finding the apparent best parameters and model), applying that learned model to the holdout set. The base learner is an SVM.

We applied the resulting learned model to the holdout set, but found that it did not perform as well as state-of-the-art methods that also only use structural MRI features. This process was still relevant, as it identifies relevant features, which will be used within LeFM_*SF*_; see Section 2.6 below.

### 2.5 Building a model from fMRI features: LeFM_*F*_

**Fig 5:**
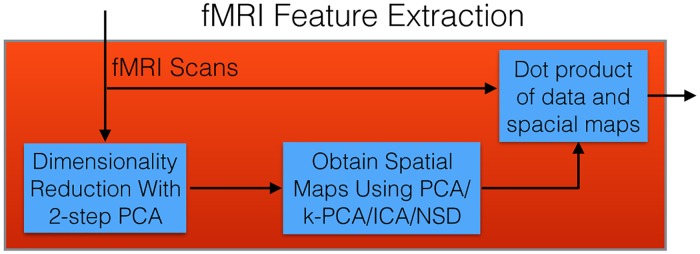
Detailed process of how a classifier is produced and used with fMRI scans. This corresponds to the “Red Box” (*i.e*., the fMRI processing component) of Fig 1.

**Fig 6:**
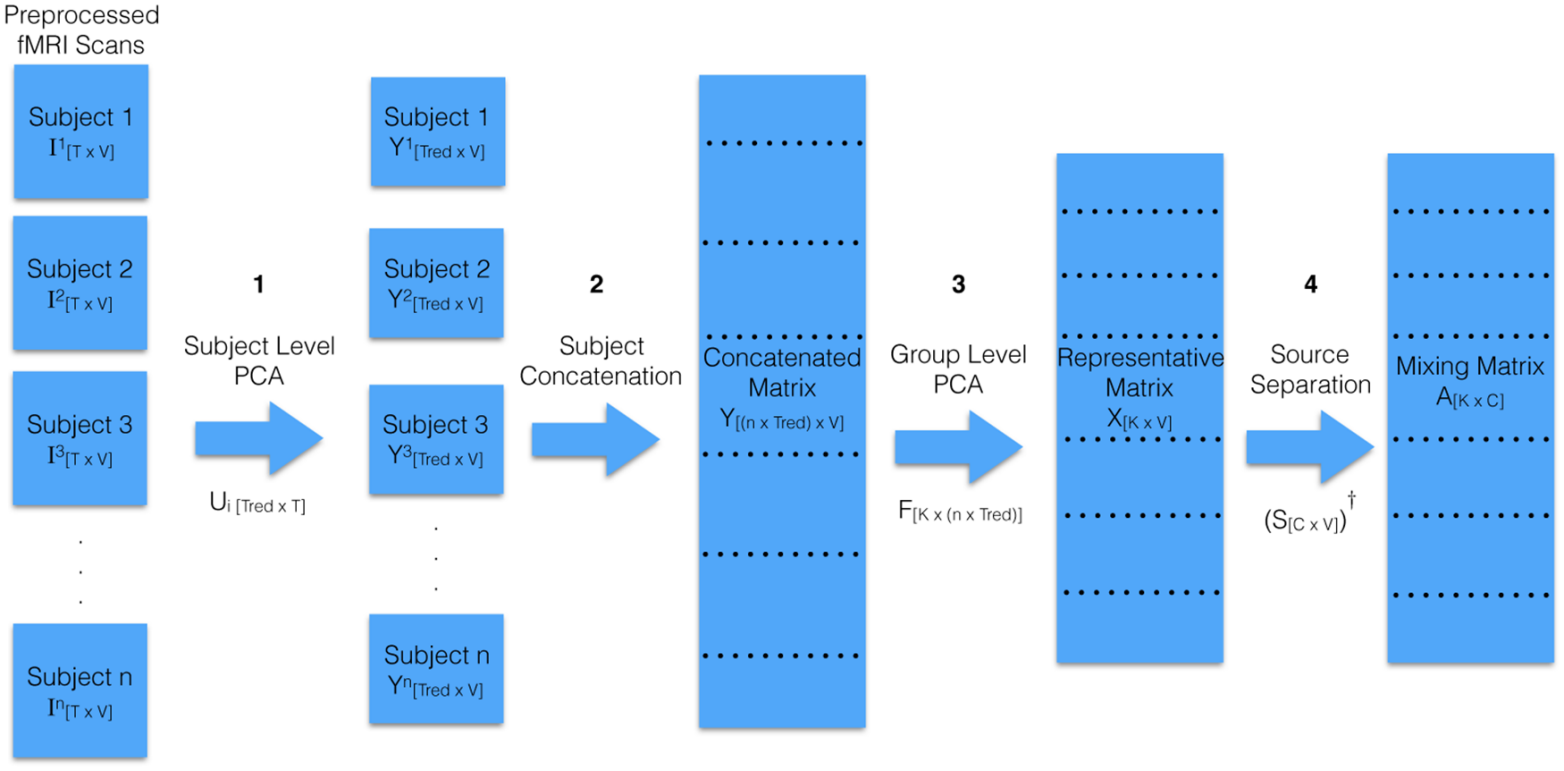
Detailed version of the fMRI data to the spatial maps process that elaborates the boxes “Dimensionality Reduction with 2-step PCA” and “Obtain Spatial maps Using PCA/k-PCA/ICA/NSD” in Fig 5. The first three of four steps show how 2-step PCA is applied to the data, where the concatenation step is temporal concatenation (Fig 7). The first column shows that the scan from subject *i* is a *T* × *V* matrix $I_{\left[T\times V\right]}^{i}$. The next column, after “Subject Level PCA”, shows the *i*^*th*^ subject is described as $Y_{\left[T_{red}\times V\right]}^{i}$, with dimension *T*_*red*_ × *V*. The subsequent “Concatentation” step produces a group matrix with dimension *nT*_*red*_ × *V*. The “Group Level PCA” step reduces this to a *K* × *V* matrix, which the “Source Separation” step factorizes to produce the mixing matrix size *K* × *C*. This final step could be the identify transformation, kernel-PCA, standard ICA or non-linear ICA; each of these might lead to different values of *C*.

This section describes our approach to producing a model that can use blind source separation to create features from a subject’s fMRI scan, where those features are then used as input to an SVM to predict the subject’s diagnosis; see Fig 5 (which expands a portion of Fig 1). Sections 2.5.2, 2.5.3 and 2.5.4 describe how we process the initial fMRI data to where source separation can be applied, then Section 2.5.5 describes the different source separation techniques that we considered, to generate spatial maps. As seen in Fig 5, we use these spatial maps to produce the fMRI-based features that we ultimately use to train an SVM classifier. Fig 6 summarizes the complete LeFM_*F*_ algorithm; the following subsections discuss each step in more detail.

As a high-level summary of the first 3 steps: an fMRI scan for a single subject has a large number of voxels, which are captured for each of a number of time points. Because combining all the fMRI scans from all subjects for source separation would be intractable, it is important to reduce the number of features while preserving the important subject-level variations in the data. To reduce the computational load on group level analysis, previous researchers have proposed various approaches for reducing the dimensionality before group level source separation analysis. We follow Calhoun *et al*. [21, 22] by using a standard 2-Step principal component analysis, applied to all fMRI images used in our study. The steps in Sections 2.5.2, 2.5.3, and 2.5.4 are implemented in the GIFT software package (http://mialab.mrn.org/software/gift/) from Calhouns’s group. This model is based on the (reasonable) assumption that there are common spatial sources for a set of fMRI scans in a particular study, where subjects differ based on temporal weights of each source. This 2-Step data reduction procedure captures the subject level variations and group level commonalities.

#### 2.5.1 Dimensionality reduction using 2-step PCA

An fMRI scan for a single subject has a large number of voxels, which are captured for each of a number of time points. Because combining all the fMRI scans from all subjects for source separation would be intractable, it is important to reduce the number of features [21, 22] while preserving the important subject-level variations in the data. In order to reduce the computational load on group level analysis, we follow Calhoun *et al*. [21]’s 2-step principal component analysis (PCA) [21, 22], which applies PCA first to each subject separately and then to the all subjects together. We use the implementation of this procedure in the GIFT software package. This 2-step PCA procedure produces a set of components meant to capture the subject level variations and group level commonalities. Each component yields a “spatial map” [23]. This approach is based on the (reasonable) assumption that there are common spatial sources for a set of fMRI scans in a particular study, where subjects differ based on temporal weights of each source.

#### 2.5.2 Subject level PCA

As shown in Fig 6, we start with a *T* × *V* matrix, $I_{\left[T\times V\right]}^{i}$ matrix describing each of the *n* subjects. *T* is the number of time points. *V* is the number of voxels. PCA [21] is used (within GIFT) to reduce this to a *T*_*red*_ × *V* matrix by selecting *T*_*red*_ largest eigenvalues, to capture 99% of the variance. (For details, see the GIFT documentation http://mialab.mrn.org/software/gift/documentation.html).

#### 2.5.3 Subject concatenation

**Fig 7:**
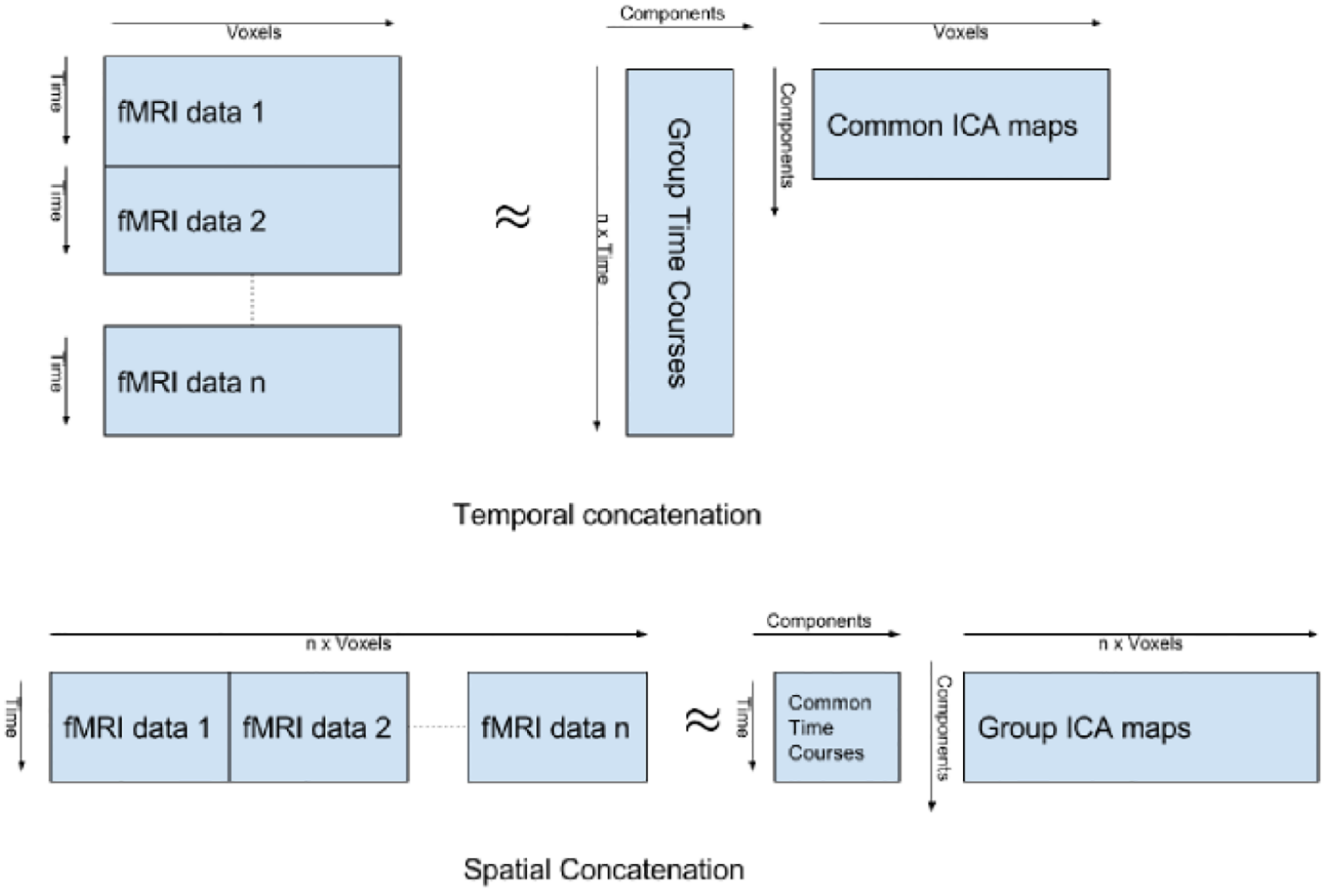
Temporal vs. spatial concatenation. Each ‘fMRI data *i*” box (for *i* = 1..*n*) corresponds to one of the *n* subjects. This shows two ways we can concatenate these boxes from subjects in Fig 6 to produce *X*, Here, we use temporal concatenation.

To estimate a set of common components across all subjects, we vertically concatenate the PCA-rotated matrices produced for each subject by the above subject-specific PCA step (as shown in Fig 7). Note that vertical concatenation means we concatenate along the temporal direction (as opposed to spatial concatenation, which is not discussed here). Therefore, we are using source separation to decompose each fMRI scan into a set of spatial maps, where each map is derived from a time course defined by a PCA component. As seen in Fig 7, in this context, the inner product of voxel components and time series components reconstructs an approximation of the original fMRI images, so we can view this as a way of representing each voxel’s time series as a linear combination of more general, shared time series.

#### 2.5.4 Group level PCA

The previous steps produced the Concatenated Matrix *Y*_[(*nxT*_*red*_)*xV*]_ (3^rd^ column in Fig 6). We next run PCA on this matrix, and again included enough rows *K* to capture 99% of the variance.

#### 2.5.5 Source separation

Previous studies [21, 24] have explored brain regions that are strongly temporally coherent (co-activated during rest) using source separation techniques like PCA or independent component analysis (ICA). Here, PCA separates the fMRI brain scan into uncorrelated spatial maps or sources based on variations in the time, whereas ICA decomposes the brain fMRI scans into a common set of spatially independent components (a.k.a. sources or spatial maps) and their corresponding user-specific time courses. In this case, ICA assumes that the spatial maps have constant higher order statistics [21, 22, 25].

This paper compares four source separation methods, which are applied after 2-step PCA—including this simple version of ICA, as well as a more complex Non-stationary Spatial Sources Decomposition (NSD) technique—in terms of the accuracy of the models produced by the features obtained from each algorithm. To the best of our knowledge, this is the first study to fully investigate using NSD to produce predictive models from fMRI data.

**Principal Component Analysis (PCA) for fMRI** The simplest of our 4 approaches just uses the “Representative Matrix” *X*_[*K*×*V*]_ as our spatial maps, without any further modification (that is, the 4th step of Fig 6 is just the identity transformation). In this case, we use internal cross-validation to determine the appropriate number of components that optimizes the accuracy of the final model, instead of preserving 99% of the variance.

**Kernel Principal Component Analysis (k-PCA) for fMRI** Here, we use the *Radial Basis Function kernel* (*RBF*), with the associated matrix,

$$\begin{array}{c} \Sigma_{RBF}\left(i,j\right)=\exp(-\frac{{\left(x_{i}-x_{j}\right)}^{T}\left(x_{i}-x_{j}\right)}{2\sigma^{2}}) \end{array}$$ (4)

where *x*_*i*_ and *x*_*j*_ are *i*^*th*^ and *j*^*th*^ column of *X* respectively. After computing the kernel similarity matrix, we can obtain the eigenvalue/eigenvector pair of the kernel matrix; the projection onto these eigenvectors produces spatial maps (rows of *S*). Finally, the inner product of each patient’s fMRI scan on these maps results in the time components (columns of *A*_*i*_). The number of eigenvectors retained for this final inner product is again selected through internal cross-validation.

**Independent Component Analysis (ICA)** The goal of the standard ICA algorithm is to find independent sources that maximize the log likelihood of the observed data, in the context of temporally concatenated fMRI data—*i.e*., given the *X* appearing in 4th column of Fig 6. We also suggest how one might interpret this process.

The standard max likelihood estimate (MLE) interpretation of ICA starts with an observed data matrix *X* where each row of *X* (*x*^*i*^) is a single observation. In particular, this model assumes that each *x*^*i*^ is a realization of the random variable **x**^*i*^ where **x**^*i*^ is a linear combination of independent source random variables, {**s**^**j**^}_*j* = 1..*K*_. We also need a mixing matrix *A* that defines the contributions of each **s**^**j**^ in producing each **x**^*i*^. Concretely, if we take any column of *X* (denoted *x*_*j*_) we find the relationship

$$\begin{array}{c} x_{j}\;=\;A\;s_{j},\;s_{j}\;\approx \;W\;x_{j} \end{array}$$ (5)

where *W* = *A*^†^ is pseudo-inverse of *A* and *s*_*j*_ is a column of *S*, the matrix whose rows *s*^*i*^ are each a realization of **s**^**i**^. Note that *A* and *S* are never observed.

**Fig 8:**
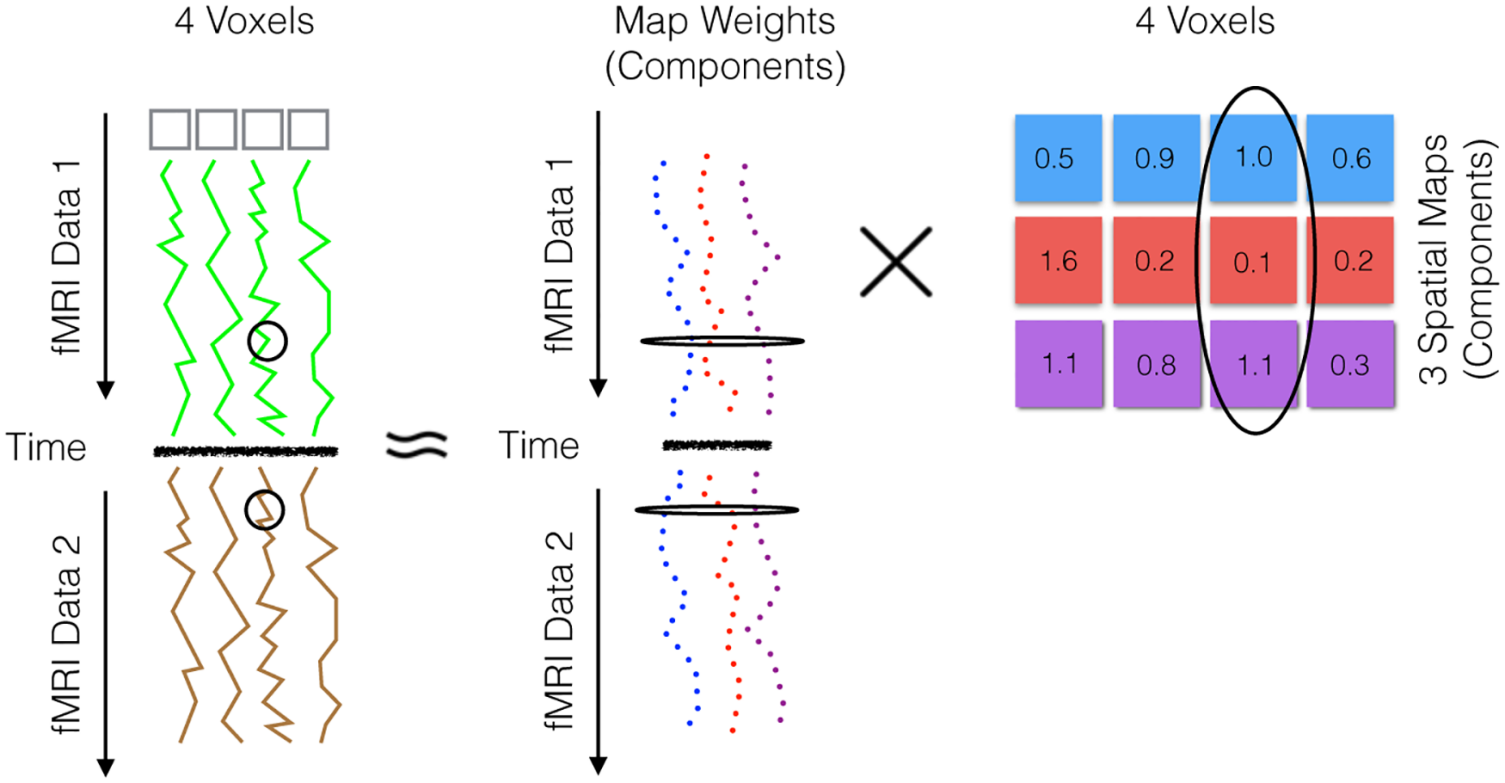
Temporal concatenation with ICA elaborated from Fig 7. This corresponds to the ICA decomposition *X* ≈ *AS*, except here, for illustrative purposes, we have replaced *X* with *Y* (the concatenated fMRI data from Fig 6 before the Group-level PCA).

In our fMRI setting, shown in Fig 8, *X* and *S* each have *V* columns and each time point in the concatenated data *x*^*i*^ is a linear combination of our unknown independent spatial maps *S* = [**s**^**1**^, …, **s**^**K**^]. To help understand ICA applied to temporally concatenated fMRI data, think of the data as snapshots of a someone performing different gestures. For each snapshot, we observe the brain activation associated with a particular gesture (our *x*^*i*^), which can be viewed as a combination of what would be the brain activations associated with more primitive movements *S* such as rotating a wrist or lifting an arm. In light of this example, ICA is being used to decompose neurological activity into more fundamental components to identify a disease state such as ADHD or Autism.

Now that we have defined our setting and provided some intuition, we now describe how we use MLE to obtain the (unobserved) independent components of *S*. Our ultimate goal is to find an optimal inverse mixing matrix W* that maximizes the likelihood of the observed data. Here, we assume that the distribution of the *h*^*th*^ source **s**^**h**^ is given by a density *p*_*h*_(**s**^**h**^), and that the joint distribution of the sources are independent of one another; *i.e*.,

$$\begin{array}{c} p\left(\;s^{1},\;s^{2},\;\ldots ,\;s^{K}\right)=\prod_{h=1}^{K}p_{h}\left(\;s^{h}\;\right) \end{array}$$ (6)

The probability of the observed signals for *j*^*th*^ observation (*x*_*j*_) is given by

$$\begin{array}{c} P\left({\left[x^{1},x^{2},...,x^{K}\right]}^{T}=x_{j}\;\right)\propto \prod_{h=1}^{K}p_{h}\left(w_{h}^{T}\;x_{j}\right)\;\times \;\text{det}\left(W\right) \end{array}$$ (7)

where $w_{h}^{T}$ is the transpose of the *h*^*th*^ column of *W* [26]. The *W* that maximizes the log-likelihood given the data *X*_[*K*×*V*]_, is

$$\begin{array}{c} W^{*}\;=\;\underset{W}{\mathrm{argmax}}\;(\sum_{h=1}^{K}\sum_{j=1}^{V}\log\;p_{h}\left(w_{h}^{T}\;x_{j}\right)\;+\log\;\text{det}\left(W\right)) \end{array}$$ (8)

Note that this *p*_*h*_(⋅) can be any non-Gaussian distribution [27]. Here, we use GIFT’s default implementation and parameters, which is

$$\begin{array}{c} p_{h}\left(\;\text{seeing}\;\text{a}\;\text{voxel}\;\text{with}\;\text{intensity}\;x\;\right)\;=\;\frac{\lambda^{\left(h\right)}}{2}\exp(-\lambda^{\left(h\right)}\;|x|) \end{array}$$ (9)

where each different source *h* = 1..*K* will have a different λ^(*h*)^. Note this does not depend on the location of the voxel. Given this W*, and the observed *x*_*j*_ for patient *j*, we then use Eq 5 to solve for the time course *A*_*j*_.

**Non-Stationary Spatial Sources Decomposition (NSD)** As mentioned earlier, the probability density of each source is parameterized by a constant value that does not change with location of voxels. NSD generalizes this, by allowing the probability density function for each component to be parameterized by the location of the voxels [28, 29]. Here, we define the distribution for the *h*^*th*^ component *S*^*h*^ as

$$\begin{array}{c} p_{h}\left(\;\text{seeing}\;\text{a}\;\text{voxel}\;\text{at}\;(\text{i},\text{j},\text{k})\;\text{with}\;\text{intensity}\;x\;\right)\;=\;\frac{\lambda_{ijk}^{\left(h\right)}}{2}\exp(-\lambda_{ijk}^{\left(h\right)}\left|x|\right) \end{array}$$ (10)

(Compare to Eq 9)

We think that each of these spatial sources should be non-stationary (*i.e*., have different λ_*ijk*_ values at different (*i*, *j*, *k*) locations) as:

1. One source (which corresponds to one row in *S* [see Fig 6]) may not have the same magnitude and variation throughout the whole brain due to in-homogeneous magnetic susceptibility that depends on the location of voxels in the scan. This differs from commonly used source separation models, which require that the sources have same variation for the whole brain scan.
2. The strength and variability within a particular source depends on the brain tissue type in a particular brain region. For example, the activation values in grey matter and white matter (which depends on the amount of oxygenated blood flow in that tissue) would be different.

In order to develop the theory for nonstationary source decomposition, consider a representative fMRI scan *X*_[*K*×*V*]_ ≈ *A*_[*K*×*K*]_ *S*_[*K*×*V*]_, where *S* is an *K* × *V* matrix of source components, where each row *S*_*r*,:_ (size 1 × *V*) specifies the contribution of voxel co-ordinates to *r*^*th*^ source. Each spatial activation refers to one row in *S*. Column *h* in matrix *A* will have the time courses for the corresponding *h*^*th*^ spatial component.

In mathematical terms, suppose we have *K* independent spatial maps (each corresponding to a row in *S*) and *V* observations (each corresponding to a 3D brain scan at a time point). Then, we can formulate the covariance matrix at location *r* = (*i*, *j*, *k*) as *R*_*x*_(*r*) = 〈*x*(*r*) *x*(*r*)^*T*^〉 = *A* *D*_*s*_(*r*) *A*^*T*^ where *R*_*x*_ amd *D*_*s*_(*r*) are of size *K* × *K*. Further we let *x*(*r*) = *X*(:, *N*(*r*)) where *N*(*r*) represents any suitably chosen region around position *r* for which the signals are assumed to have same higher order statistics. For our experiments, we have chosen *N*(*r*) to be a 4 × 4 × 4 bounding box (*e.g*., if the point r = [200, 100, 50], then this box is defined by corners [199, 99, 49] and [202, 102, 52]). We found, empirically, that increasing the bounding box severely degraded the performance of the model.

Assuming the sources are spatially non-stationary, and following [15, 30],

$$\begin{array}{ccc} x(r) & \approx & A\;s(r)\\ R_{x}\left(r\right)\;=\;\left\langle x\left(r\right)\;x{\left(r\right)}^{T}\right\rangle & = & A\;\left\langle s\left(r\right)\;s{\left(r\right)}^{T}\right\rangle \;A^{T}\;=\;A\;D_{s}\left(r\right)\;A^{T} \end{array}$$

However, as we do not have a perfect estimate for *R*_*x*_(*r*), we estimate the covariance matrix *R*_*x*_(*r*) for some spatial interval. We denote the sample estimates as ${\hat{R}}_{x}\left(r\right)$, and define the measurement error as

$$E\left(r\right)\;=\;{\hat{R}}_{x}\left(r\right)\;-\;A\;D_{s}\left(r\;\right)A^{T}$$

Suppose we have *N* samples of ${\hat{R}}_{x}\left(r\right)$ for *r* ∈ {*r*_1_, *r*_2_, .., *r*_*N*_}, then we can estimate the parameters by

$$\hat{A},\;{\hat{D}}_{s}\left(r_{1}\right),\;{\hat{D}}_{s}\left(r_{2}\right),\;\ldots ,\;{\hat{D}}_{s}\left(r_{N}\right)=\underset{A,D_{s}\left(r_{1}\right),...,D_{s}\left(r_{N}\right)}{\mathrm{argmin}}\sum_{k=1}^{N}{\left\|E\left(r_{k}\right)\right\|}^{2}$$

and estimate the source components as

$$\hat{s}\;=\;\underset{s}{\mathrm{argmin}}{\left\|x-\hat{A}s\right\|}^{2}$$

Then for each patient, we can calculate the time courses *A*_*i*_ from Eq 11. This method decorrelates the spatial sources at different regions of the brain, which is desirable for the reasons described above.

#### 2.5.6 Complete LeFM_*F*_ process

Fig 6 shows the steps. First, the SubjectLevelPCA reduces each subject’s [*T* × *V*] matrix *I*^*i*^ to *Y*^*i*^ = *U*_*i*_*I*^*i*^ where *U*_*i*_ is the *T*_*red*_ × *T* reduction matrix. Note that *T*_*red*_ is determined by retaining the independent components that preserve 99% of the variance (see the GIFT software package documentation for complete details on individual and group level PCA reductions). The second step, Concatenation, concatenates {*Y*^*i*^}_*i* = 1..*n*_ over all of the *n* subjects, to obtain the matrix *Y* of size *nT*_*red*_ × *V* The third step, GroupLevelPCA, runs PCA on this *Y* to produce a new matrix *X* ≈ *FY*, where *F* is a *K* × *nT*_*red*_ reduction matrix. If we divide the *F* into *n* sub-blocks *F*_*i*_, each of size *K* × *T*_*red*_, then *F*_*i*_ corresponds to reduction matrix for subject *i*. We then consider various final steps: (1) Directly using the final *F* matrix as features. (2) Running kernel PCA on *X* to obtain a reduction matrix *A*. (3) Running (stationary) ICA on *X* to obtain the mixing matrix *A*. (4) Running NSD on *X* to obtain the mixing matrix *A*.

In these last three variants we are using different blind source separation algorithms to express *X* ≈ *A* × *S*, where *S* is a [*C* × *V*] matrix with *C* spatial maps covering *V* voxels. Hence, for subject *i*,

$$\begin{array}{c} X^{i}\;=\;U_{i}^{†}\;F_{i}^{†}\;X\;\approx \;U_{i}^{†}\;F_{i}^{†}\;A\;S\;=\;A_{i}\;S \end{array}$$ (11)

where $A_{i}\;=\;U_{i}^{†}F_{i}^{†}A$, which is different for each patient (while all use the same *S* matrix). Here $U_{i}^{†}$ and $F_{i}^{†}$ are pseudo-inverses of *U*_*i*_ and *F*_*i*_ respectively. Thus, *X*^*i*^ is approximated as *A*_*i*_ *S*.

### 2.6 Building a model from multimodal features: LeFM_*SF*_

For the set of all *n* subjects, let $X_{n\times f1}^{mri}$ be the feature matrix from the one-layer convolutional network on the MRI data, and $X_{n\times f2}^{fmri}$ be feature matrix from applying nonstationary ICA to the fMRI data. The combined feature matrix is $X_{n\times f}^{combined}$, with *f* = *f*1 + *f*2 columns. This matrix was then divided into training set and holdout set by instances as in Section 2.1. The training set was then used to train a linear support vector machine.

### 2.7 Support vector machine classifier

Each of the approaches—LeFM_*S*_, LeFM_*F*_, LeFM_*SF*_—uses the Support Vector Machine (SVM) learning algorithm to generate their models, based on the respective features. LeFM_*S*_ and LeFM_*SF*_ use linear kernels and LeFM_*F*_ uses an RBF kernel. This is based on the argument that when we use linear features (*i.e*., features are extracted using a linear transform) we use a non-linear classifier, and when we use non-linear features (*i.e*., features are extracted using a non-linear transform) we use a linear classifier. For the RBF kernel, we follow a standard practice of using internal cross-validation to set the parameter *γ* ∈ {0.1 × 2^*i*^|*i* = 0..10}. The parameter *C* for all SVMs is left at the default of *C* = 1. Of course, once the model is produced by the learner, it can then be used to predict the class of a novel instance.

## 3 Results

This section provides the results of cross validation and holdout set evaluation for the three models considered: Section 3.1 discusses the results of the LeFM_*S*_ model; Section 3.2 discusses the results of the LeFM_*S*_ model; and Section 3.3 discusses the results of the LeFM_*SF*_ model. We see that our LeFM_*S*_ model is not better than state-of-the-art MRI based models, in general, but our LeFM_*F*_ model outperforms other models that only use fMRI features, and our LeFM_*SF*_ model outperforms all other image-based models, on this dataset.

### 3.1 Results using MRI texture-based features (LeFM_*S*_)

The best 5-fold cross-validation accuracy achievable for ADHD-200 data is 0.635. When run on the hold-out data, this classifier yielded accuracy of 0.626, with sensitivity, specificity and Jstat of 0.420, 0.842 and 0.262 respectively. The best 5-fold cross-validation accuracy achievable for ABIDE data is 0.614. When run on the hold-out data, this classifier yielded accuracy of 0.617, with sensitivity, specificity and Jstat of 0.490, 0.730 and 0.220 respectively. Here, cross validation found that the optimal number of spatial maps for ADHD was 100, and for ABIDE, it was 175.

### 3.2 Results using fMRI source separation models (LeFM_*F*_)

#### 3.2.1 5-fold cross validation accuracy

**Fig 9:**
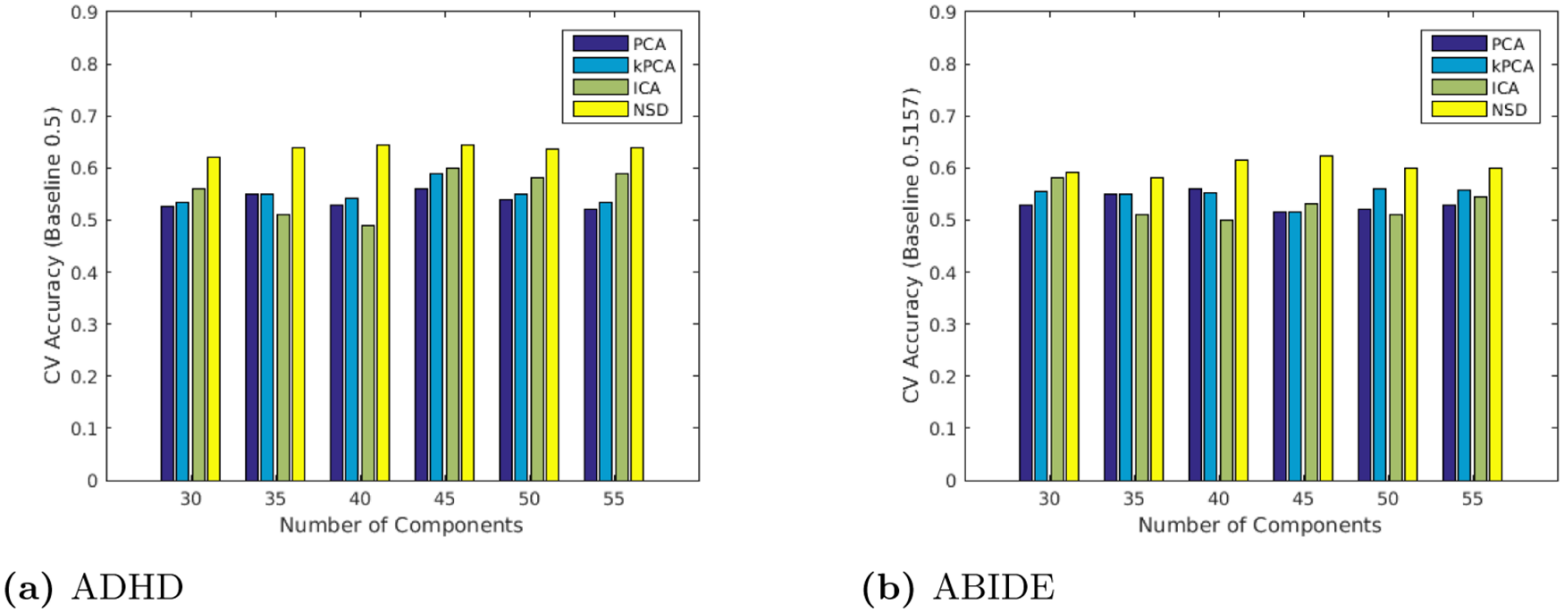
5-fold cross validation results of classification.

To assess the predictive ability of a model trained only on fMRI features, we ran 5-fold cross validation (CV) over a varying number independent components. We then ran the version with the optimal number on the holdout set. Table 3 shows the results of this CV selection process for both the ADHD and Autism datasets. As shown, for the ADHD data, the model performed best with 45 independent components, achieving an accuracy of 0.6450 compared to the baseline of 0.50 with significance p = 1.83e-12 (via binomial test). For the Autism data, the model performed best with 45 components with a CV accuracy of 0.6225 relative to the baseline 0.5157 (p = 4.95e-10, via binomial test). Note that the 45 components for the ADHD data are unrelated to the 45 components for the Autism data. Fig 9 plots these values, and shows that NSD is better than the other feature extraction methods (PCA, kPCA and ICA).

**Table 3:** 5-fold CV results for classification accuracy (and standard deviation) using various numbers of independent components.

	# ICs (SVM GAMMA)	5-Fold CV Accuracy	5-Fold CV STD
ADHD	30 (6.4)	0.6216	0.0399
	35 (3.2)	0.6378	0.0374
	40 (1.6)	0.6432	0.0390
	45 (3.2)	**0.6450**	0.0291
	50 (3.2)	0.6360	0.0261
	55 (3.2)	0.6378	0.0075
Autism	30(51.2)	0.5925	0.0158
	35 (51.2)	0.5825	0.0049
	40 (51.2)	0.6159	0.0078
	45 (51.2)	**0.6225**	0.0210
	50 (51.2)	0.6000	0.0130
	55 (51.2)	0.5987	0.0059

Note: SVM GAMMA refers to the RBF kernel variance parameter $\gamma =\frac{1}{2\sigma^{2}}$.

#### 3.2.2 Model hold-out test accuracy

Using the optimal parameters selected from cross validation, the resulting hold-out accuracy for ADHD was **0.6491** (baseline 0.5497) and was **0.6233** for ABIDE (baseline 0.5157). The specificity, sensitivity and Jstat for ADHD (resp., ABIDE) are 0.8191, 0.4416 and 0.2607 (respectively, 0.6768, 0.5533 and 0.2301). To our knowledge, our holdout accuracies achieved on ADHD-200 test data and the ABIDE test data are the best known when only using fMRI scans.

#### 3.2.3 Leave-one-out accuracy comparison

To compare our results with yet other systems, we also computed the leave-one-out accuracy of our ABIDE model; Table 4 shows that our LeFM_*F*_ was superior.

**Table 4:** Leave-one-out results for autism classification (ABIDE) using only imaging data.

Algorithm	Accuracy	Specificity	Sensitivity	J-Statistics
LeFM_*F*_	**0.614 ±0.017**	0.648	0.578	0.226
Nielsen et al [14]	0.600 ±0.016	0.58	0.62	0.20

Note that Abraham *et al*. [31] report an “inter-site” cross-validation accuracy of 66.8±5.4% on a version of the dataset containing 871 subjects (patients are ommited because they did not pass a visual quality inspection). Here, Abraham *et al*. [31] use a 16-fold cross validation, done by training on 15 of 16 sites and testing on the remaining site in each fold. By contrast, our model was trained on a smaller set of only 800 instances—recall we trained on only 70% of the original 1111 instances, and left the remaining 30% (311) for testing. It is likely that their reportedly high accuracy is due in part to having a larger CV dataset where samples were heavily scrutinized so as to exclude those that did not meet their criteria.

#### 3.2.4 Spatial maps

#### *Spatial maps from ADHD-200 data*:

**Fig 10:**
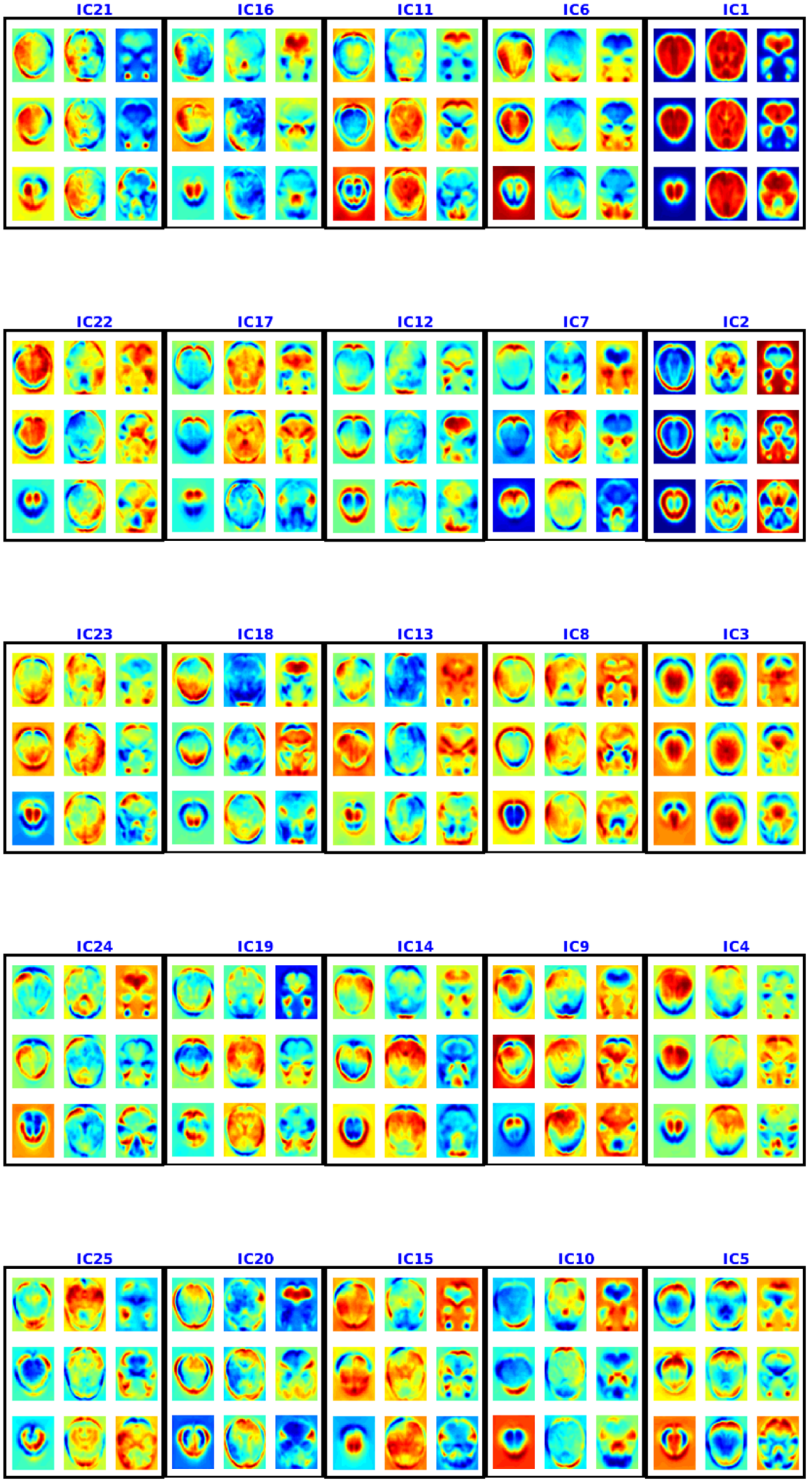
Spatial maps (aka components) 1–25 for ADHD-200 using NSD. Each 3×3 box shows 9 axial slices of one component. The colorbar is same as Fig 4.

**Fig 11:**
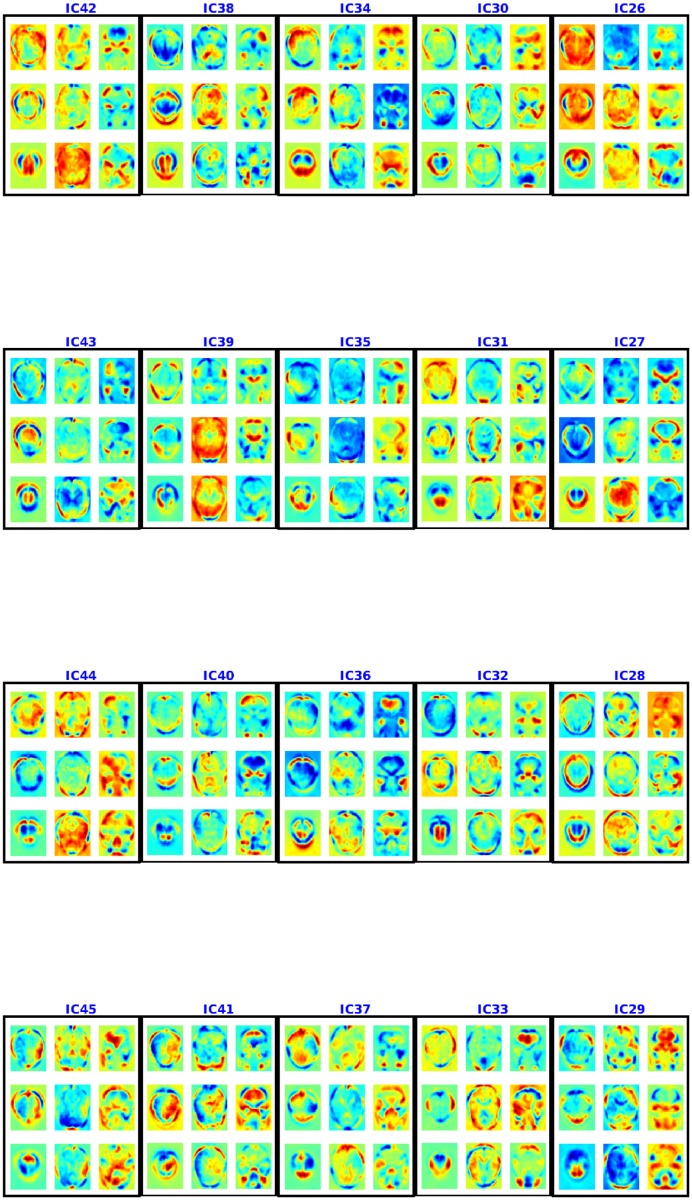
Spatial maps or components 26–45 for ADHD-200 using NSD. Each component is shown in a box and 9 axial slices are shown. The colorbar is same as Fig 4.

Figs 10 and 11 show the spatial maps found using multiple de-correlation—showing 9 axial slices for each. The component IC1 appears to be an artifact as it assigns very high values to all the voxels—*i.e*., as all the voxels in the brain are approximately of equal importance in this component, it is shared by all the voxels. We assume this is probably due to noise like motion, breathing and attention signals, which can modulate voxels throughout the brain [32]. We believe that IC3 also appears to be an artifact as it consists of regions from cerebrospinal fluids which means it might be a result of cardiac pulsatility artifacts [33]. Moreover, we found that removing these two artifacts did not change the performance of the model. The other components consist of some common default mode networks (regions that are shown to be active during rest [34]) and apparently some new resting state networks not previously identified in the literature. Some of the resting state networks found are consistent with [35]. For example, IC5 appears similar to the resting state network for peristriate area, and lateral and superior occipital gyrus, which are areas related to visual cortex and might represent spontaneous brain activities like day-dreaming [36, 37]. It is easy to connect this component to ADHD as ADHD patients are more likely to experience mind-wandering [38]. IC6 captured shared functional properties in the frontal and occipital lobe (responsible for planning and many areas of vision respectively). This area is very important for ADHD as well, as ADHD patients may suffer from lack of effective planning [39]. IC24 consists of pons (responsible for eye movement, sleep, and many other vegetal and automatic functions) regions and temporal lobe (for sensory processing, memory formation and higher order association processing). Their usefulness in prediction suggests that some of the physical signals captured by fMRI may also be indicative of a disease state.

#### *Spatial maps from ABIDE data*:

**Fig 12:**
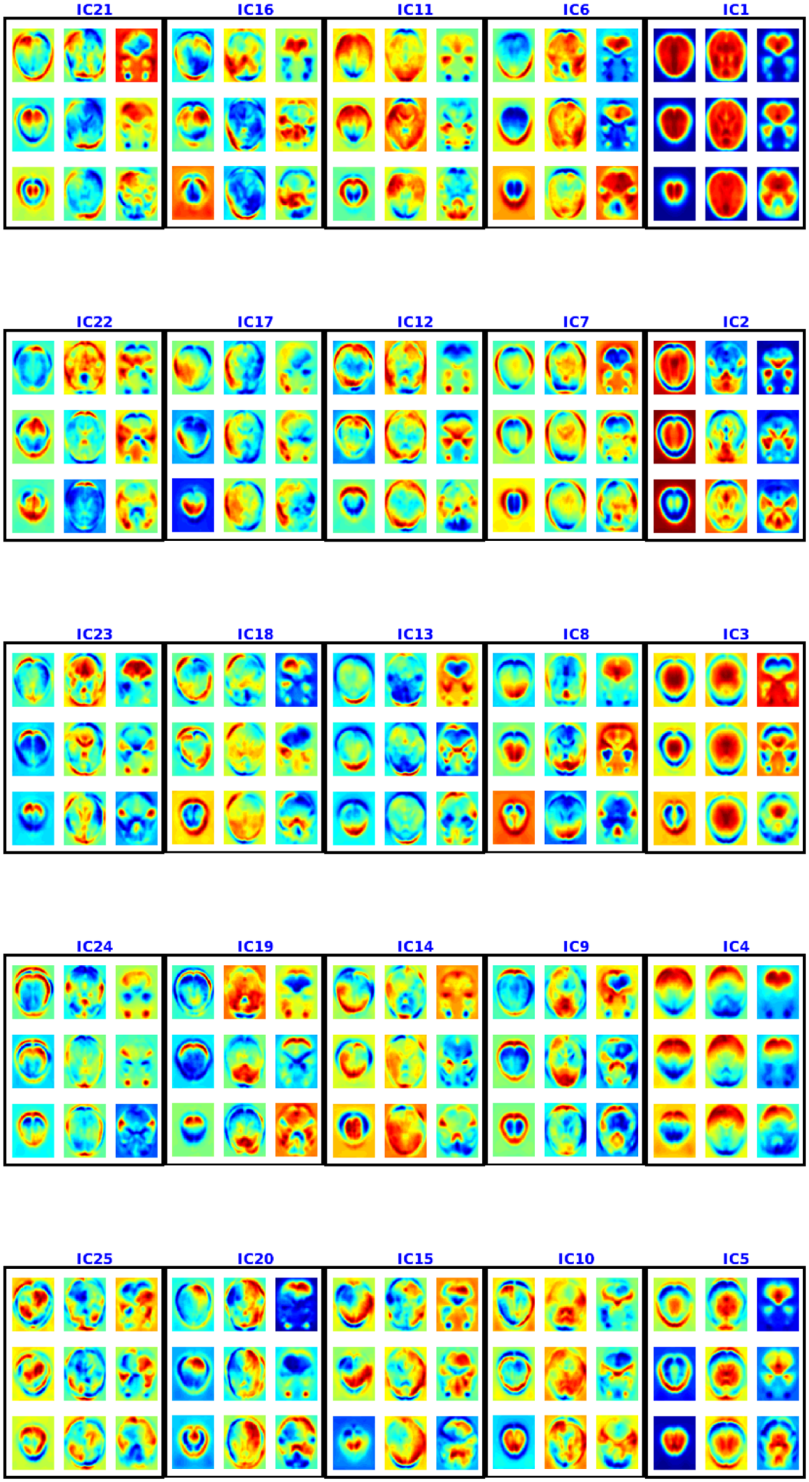
Spatial maps or components 1-25 for ABIDE using NSD. Each component is shown in a box and 9 axial slices are shown. The colorbar is same as Fig 4.

**Fig 13:**
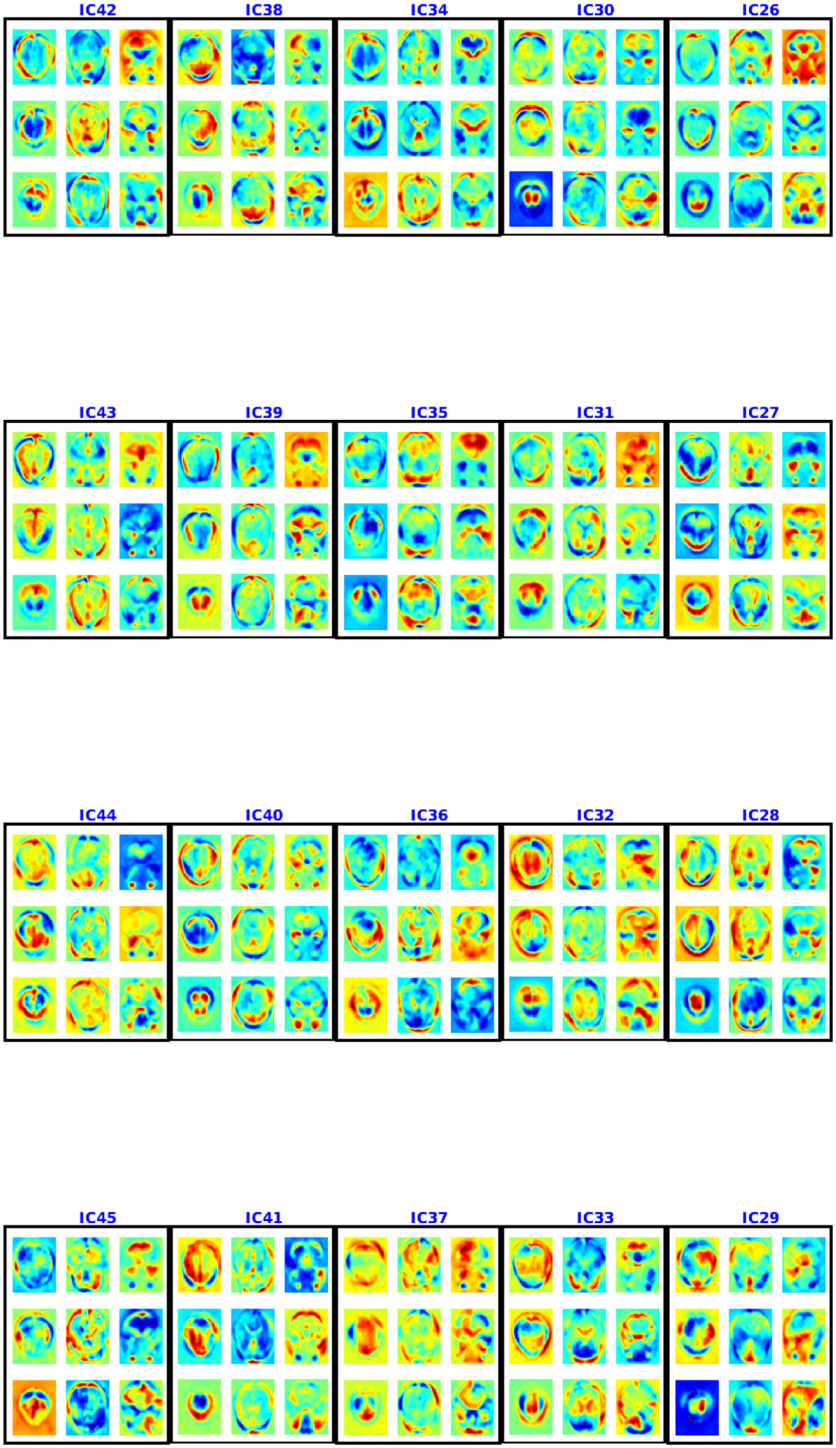
Spatial maps or components 26-45 for ABIDE using NSD. Each component is shown in a box and 9 axial slices are shown. The colorbar is same as Fig 4.

Figs 12 and 13 present the spatial maps found using multiple de-correlation. This IC1, too, appears to be an artifact as it is shared by almost all the voxels [32]. IC3 also appears to be an artifact as it consists of regions from cerebrospinal fluids and is a result cardiac pulsatility artifacts [33]. A deeper investigation into the components shows multiple overlapping components. The components include visual areas (visual cortex, V1 and V2), partial overlapping with some default mode networks (PCC/precuneus, anterior cingulate cortex and frontal lobe) and motor networks. These components are informative: see the cross-validation, test accuracy on the ABIDE data for autism classification.

### 3.3 Results using multi-modal features (LeFM_*SF*_)

Table 5 shows the accuracy of the LeFM_*SF*_ learner for both ADHD-200 and ABIDE; these values shown the best known using only imaging data. (That table also shows the hold-out specificity, sensitivity and Jstat values for the datasets.) Table 6 summarizes the results of this model, as well as our other models, in comparison to previous methods.

**Table 5:** LeFM_*SF*_ results for ADHD/ABIDE classification—using structural and functional scan features.

	5-CV	Hold-Out			
	Accuracy	Accuracy	Specificity	Sensitivity	Jstat
ADHD	0.6892	0.6725	0.8510	0.4545	0.3055
ABIDE	0.6312	0.6431	0.6832	0.6000	0.2832

**Table 6:** Hold-out test results for ADHD classification using only fMRI and/or MRI features.

Data	Type	Algorithm	Accuracy	Specificity	Sensitivity	J-Statistics
ADHD	fMRI/MRI	LeFM_*SF*_	**0.673**	0.851	0.455	**0.306**
ADHD	MRI	Ghiassian et al [40]	0.661	0.545	0.755	0.300
ADHD	fMRI	LeFM_*F*_	0.649	0.819	0.442	0.261
ADHD	MRI	LeFM_*S*_	0.626	0.842	0.420	0.262
ADHD	fMRI	Ghiassian et al [13]	0.626	_	_	_
ADHD	fMRI/MRI	Dai et al [11]	0.615	0.7766	0.4133	0.1833
ADHD	fMRI	Sidhu et al [12]	0.614	_	_	_
ADHD	fMRI	Eloyan et al [10]	0.610	**0.94**	0.21	0.15
ADHD	fMRI	Ghiassian et al [40]	0.597	0.299	**0.840**	0.139
ABIDE	fMRI/MRI	LeFM_*SF*_	**0.643**	0.683	0.600	**0.283**
ABIDE	fMRI	LeFM_*F*_	0.623	0.677	0.553	0.230
ABIDE	fMRI	Ghiassian et al [13]	0.619	_	_	_
ABIDE	MRI	LeFM_*S*_	0.617	**0.730**	0.490	0.220
ABIDE	MRI	Ghiassian et al [40]	0.601	0.491	0.704	0.195
ABIDE	fMRI	Ghiassian et al [40]	0.592	0.454	**0.722**	0.176

### 3.4 Quantifying significance

**Fig 14:**
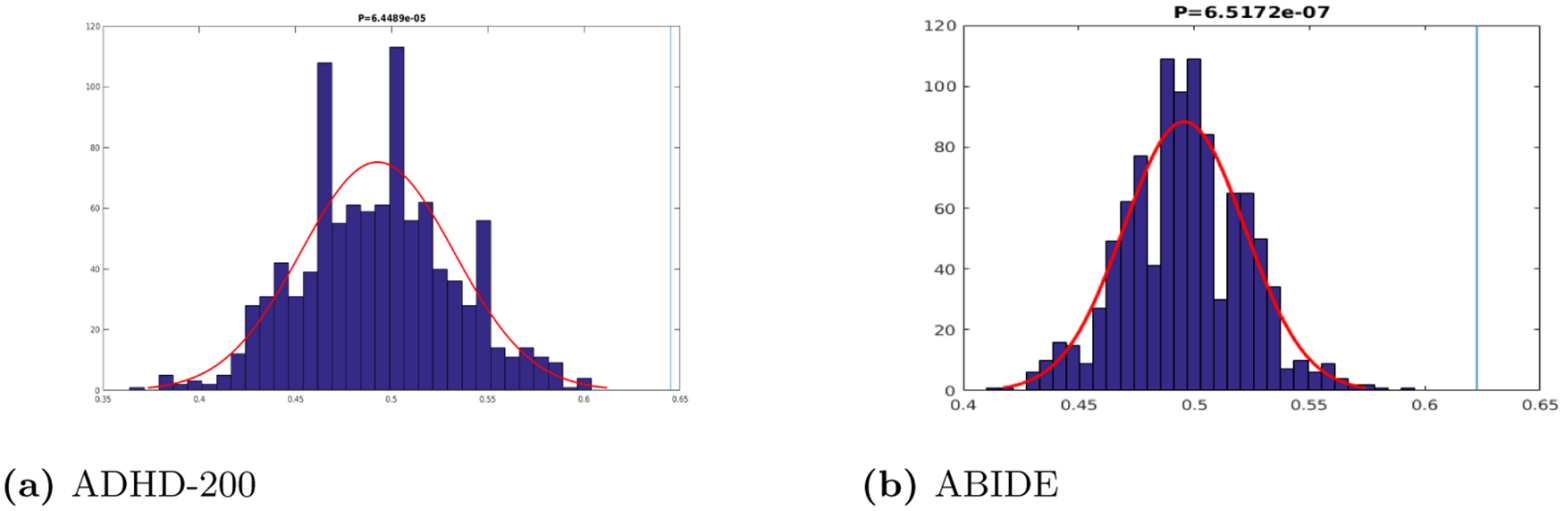
Permutation tests.

To further establish that our results are better than chance, we performed *permutation tests*. This involves computing a trivial baseline—the accuracy produced if there was “no signal” between the features and label—then determining if our learned model performed significantly better than that. Here, for each dataset (ADHD-200 and ABIDE), we performed 1000 iterations: each time, we randomly permuted the subject labels to effectively remove any relationship between the input features and the label, then we trained a model on the training subset of this set and tested it on the remaining subset. Fig 14 shows the distributions of accuracy scores for the two datasets. In each case, we see that there is a significant difference (ADHD-200 p-value = 6.45e-5 and ABIDE p-value = 6.52e-7) between the centers of the distributions and the accuracy obtained by the LeFM_*SF*_ model.

In addition to the permutation tests, we also performed four McNemar tests to determine if LeFM_*SF*_ significantly out-performed our other models, LeFM_*S*_ and LeFM_*F*_Ẇe found that LeFM_*SF*_ had significant improvements over LeFM_*S*_ for the ABIDE data (p-value = 0.038) but not for the ADHD (p-value = 0.806) data. However, there were no significant improvements for LeFM_*SF*_ over LeFM_*F*_ for ADHD-200 (p-value = 0.808) or ABIDE (p-value = 0.602). While the other tests were not *p* < 0.05 significant, note that LeFM_*SF*_ always performed better than either LeFM_*F*_ or LeFM_*S*_ on those hold-out sets (for both ADHD-200 and ABIDE). Moreover, LeFM_*SF*_ had better performance in terms of cross-validation training accuracies. Note also that this particular difference is not a major part of our claims—*i.e*., our major contribution is demonstrating that applying NSD to fMRI images can facilitate psychiatric diagnosis, for two different disorders.

### 3.5 Site specific effects

We observed that the proportion of cases versus controls varies across the sites—with some sites being predominantly cases while others were mostly controls. We therefore considered the possibility that our learned classifier may have picked up some site-specific difference (unrelated to disease) and used that as a proxy for the label—in effect, perhaps, simply setting everyone in the *Oregon* subset to be “Control” (the case:control ratio was 6:28 for Oregon). As this site-identifying-proxy is clearly not biologically relevant, the classifier is unlikely to be accurate for other subjects, especially ones from yet other sites.

We tested this in two ways: First, to see how influential site information could be, we described each patient based only on his/her site IDs (one hot encoded). We then trained a linear model to predict the subject’s disease status given only that site information. Note that our earlier learners did not use the site information—*i.e*., our description of each subject did not include the “site” feature. We found that models trained using only site information achieved an accuracy of 66.0% on the ADHD data and 55.0% on the ABIDE data, while our models trained on MRI data achieved much higher accuracies: 67.4% and 64.3%, respectively.

Second, we realized that that a “site-biased” classifier would perform badly if run on a new test sample that was balanced on each site (*i.e*., which included 50% cases and 50% controls). For example, if a classifier simply identified which subjects were from the *Oregon* subset, and set them all to “Control”, this classifier would only be 50% accurate for this 50:50 testing subsample. To test this possibility, we created new “site-balanced test sets” (for both the ADHD-200 and ABIDE datasets) by subsampling from the original test sets such that the case:control ratio was essentially 50:50 for each site. We then tested those previously-trained classifiers on these new “site-balanced test sets” and found that the accuracies did not differ significantly from the original test set results: here, it was 69.88% for ADHD-200 and 63.46% for ABIDE. Moreover, its accuracy for Oregon was 68%.

## 4 Discussion

### 4.1 Spatial sources decomposition

In general, an ICA model uses the individual time courses for each component as features for the learner. Here, the separation model for the *i*^*th*^ subject is $X_{\left[T\times V\right]}^{i}\;\approx \;A_{\left[T\times I\right]}^{i}\times S_{\left[I\times V\right]}$ where rows of *S* are estimated spatial map and columns of *A* are corresponding estimated time courses. The time course components *A*_:,*h*_ correspond to weighting of the component *S*_*h*,:_, where a smaller component weight *A*_:,*h*_ corresponds to lower contribution of the component to the whole scan and indicate hypo-connectivity. Likewise higher weights for a component will represent hyper-connectivity.

For ADHD, different studies have reported different pathological changes in brain [2]. Tian *et al*. [3] showed a higher level activity in sensory cortex. Using a similar method, Castellanos *et al*. [4] conceptualized a lower connectivity between anterior cingular cortex, precuneus and posterior cingulate cortex. In our studies, we found differences in group level mean connectivities for ADHD cases for visual and default mode components corresponding to regions identified as relevant by [2, 4]: *S* = { IC4, IC7, IC9, IC28, IC36, IC38, IC42, IC43 }. We compared corresponding group level mean weights for Healthy and ADHD patients. For IC*h* ∈ *S*, the weights *A*_:, *h*_ are reduced for ADHD patients in a total of 42, 42, 42, 42, 40, 41, 43, 36 time points out of total number of time points 91. The reduced differences were statistically significant (*p* ≤ 0.05) for many of these time points: 21, 20, 20, 21, 19, 19, 22, 18 (resp.). This suggests that the patients suffering from ADHD might have a combination of hyper-connective or hypo-connective brain, depending on time points.

For Autism, the main connectivity loss is noted in frontal lobe and other cortical areas [5, 6]. In our analysis, this corresponds to IC4, IC6, IC7, IC8, IC15, IC16, IC29, IC42, IC41. In all these cases, similar to ADHD, we compared corresponding group level mean weights for Healthy versus Autism patients. The weights are reduced for autism patients in a total of 51, 57, 50, 57, 53, 53, 52, 53, 52 time points whereas total number of time points is 91. Also these differences were significant with (*p* ≤ 0.05) for 21, 20, 20, 23, 19, 23, 20, 18, 19 of these time points. IC3 predominantly represents CSF (in ventricles in the brain). Our study shows that this component does not have much effect between healthy and autistic brains as there is group level differences (*p* ≤ 0.05) in only 18 time points—*i.e*., only 0.2 of the whole time points of IC3 are significantly different.

As shown in [13], texture based features from fMRI and MRI scans can be predictive of psychiatric diseases. Our model derives the texture based features from MRI and combines them with resting state information from fMRI to produce a strong predictor.

### 4.2 Comparison with previous results

Our LeFM_*SF*_ model performs better than any other known model on both fMRI and MRI/fMRI features, for two different datasets. However, these models only increase the prediction accuracy by 1.2% for ADHD (p = 0.909) and 4.2% (p = 0.321) for Autism, compared to the previous works on ADHD/Autism prediction. There are several possible reasons why its performance is not yet better: (1) Perhaps the resting state network structures are not significantly different between ADHD/Autism patients versus healthy controls. The previous section showed that almost half of the total number of time points for each spatial component had no statistical differences between healthy and ADHD/Autism positives.

(2) Although we applied several pre-processing steps to the data before running the machine learning process, the datasets suffer significantly from variations that depend on the site. Through our experimentation we observed that the first few principal components among the fMRI scans account for most site-dependent variations—batch effects. However a model learned without these first few components loses its predictive quality, indicating that the first few important principal components are also important in disease classification. We can use the fMRI ICA features to learn a classifier that is able to predict the *site* of each subject, with 92% accuracy. This suggest that ADHD/Autism predictability is also interlinked with site-dependent fMRI scan features—*i.e*., there are some common features that can predict both the site and the disease. While these two factors should be decoupled before we can do a true analysis of ADHD/Autism predictability using fMRI features, our analysis is an important step towards an automated generalized prediction model for psychiatric disease detection.

## 5 Conclusion

The development of automatic ADHD/Autism diagnostic algorithms from MRI/fMRI data is a challenging task. While the current results are not yet clinically relevant, these learning algorithms have found a “signal” in this data, to show that there are differences that can distinguish between case versus control. These positive results nicely motivate further research directions, to further improve these results and search for discriminative features for classifying ADHD/Autism amongst the plethora of voxel values present in structural (MRI) and functional neuroimages (fMRI): (1) exploring ways to separate sources based on deep belief networks [41]; (2) exploring yet other modalities (Diffusion Tensor Imaging, Electroencephalogram, etc.); (3) developing other methods to further reduce “batch effects”—*i.e*., minimize site-pecific influences in the data.

In this work, we derived a novel algorithm for combining structural and functional features using 3-D texture based and independent component analysis of the whole 4-D fMRI scan, which can then be used to differentiating Healthy vs Psychiatric patients, for two different diseases. We also explored different representation of brain functional connectivity useful for this classification task. Our results indicate that combining multimodal features (both MRI and fMRI) yields the best known classification accuracy for distinguish case (ADHD or Autism) from healthy controls, which is an important step towards automated computer-aided diagnosis of these (and perhaps other) psychiatric diseases.
